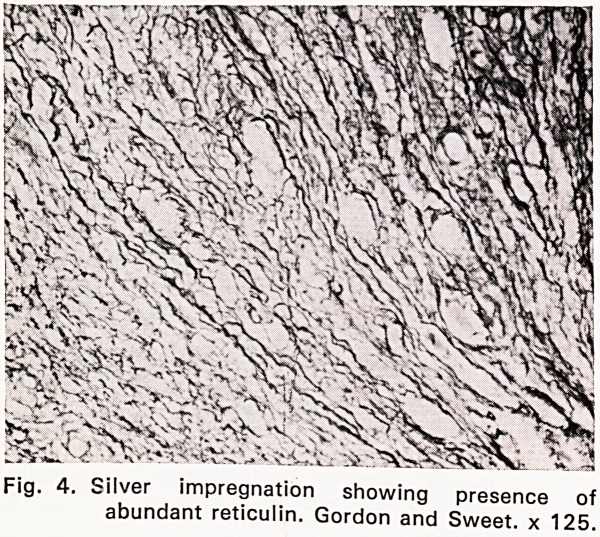# Primary Hodgkin's Disease of the Rectum

**Published:** 1975-01

**Authors:** R. Salm, C. M. Vickery

**Affiliations:** Royal Cornwall Hospital (Treliske), Truro, Cornwall; Royal Cornwall Hospital (Treliske), Truro, Cornwall


### Primary Hodgkin's disease of the rectum

**Seven-year cure after Surgical excision**

**R. SALM M.D., F.R.C.Path.**
**C. M. VICKERY F.R.C.S.**

Royal Cornwall Hospital (Treliske),
Truro, Cornwall

In disseminated Hodgkin's disease the gastro-intestinal canal may eventually become involved, though rectal involvement is unusual. Primary Hodgkin's disease of the rectum, however, is a great rarity. For this reason we wish to record such a patient who has now survived rectal excision for 7 years and who is well and apparently free from disease.

### CASE REPORT

D.A.C., a married woman aged 55 years, was first seen in September 1967 with a one-year-history of discomfort on defaecation, a slight but increasingly blood-stained mucous rectal discharge and latterly a feeling of incomplete bowel emptying. There were no general symptoms such as anorexia, loss of weight, pruritus or pyrexia. Rectal examination revealed a firm ulcerated mass, but otherwise clinical examination was negative and she was provisionally diagnosed as a case of carcinoma of the rectum. A haemoglobin concentration of 86 per cent and an ESR of 28 mm after one hour were the only minor haematological abnormalities found, all chemical blood analyses were within the normal range and a chest radiograph was normal. Sigmoidoscopy showed an extensive mobile ulcer on the right posterior wall of the lower rectum. On taking material for biopsy it was noted that this was a little more difficult than is usual in cases of carcinoma. A histological diagnosis of Hodgkin's disease was made. No lymphadenopathy was demonstrable, a sternal marrow examination showed normal marrow and a psittacosis/lymphogranuloma venereum complement fixation test was negative. Aorta-iliac lymphangiography was not considered and, in retrospect, it is doubtful if outlining the systemic lymphatic system would have been helpful in a lesion originating in the rectum. An abdomino-perineal resection of the rectum was carried out; recovery was uneventful. Postoperatively the patient did not receive any oncolytic treatment or radiotherapy. She has been followed up at half-yearly intervals with periodic chest radiographs and haematological examinations, all of which have been normal. She remains symptom-free and her weight is steady.

**Operation specimen.** This consisted of an anal rim together with 35 cm of rectum and adjacent sigmoid colon. Three eroded tumour plaques were present, one adjoining the dentate line and the other two several cm above it, measuring respectively 4.5 x 3.5 cm, 4 x 3 cm and 3 x 2 cm, and being about 0.3 cm thick. A number of soft congested lymph nodes were present along the haemorrhoidal vessels.

**Microscopical examination** (67/3613). Blocks were taken from each of the three rectal plaques. All sections showed superficially eroded tumour tissue, mainly replacing mucosa and submucosa, but occasionally also involving the internal muscle coat. The neoplastic tissue was pleomorphic and was composed of reticulum cells, lymphocytes, plasma cells, small number of eosinophils and, in some areas, very numerous bizarre, multinucleated tumour giant cells with up to a dozen vesicular nuclei (Figs. 1, 2 and 3). Only a very occasional giant cell of Sternberg-Reed type was noted. Silver stains demonstrated the presence of a fairly dense reticulin network (Fig. 4). In some areas the neoplastic reticulum cells showed pronounced mitotic

Fig. 1. Edge of malignant ulcer. From left to right mucosa, pleomorphic tumour with giant cells, necrotic exudate. Haematoxylin and eosin. x 125.

Fig. 2. High-power view of pleomorphic neoplasm. One giant cell in mitosis. H.E. x 500.

Fig. 3. A different field with unusual multinucleated giant cell. H.E. x 500.

Fig. 4. Silver impregnation showing presence of abundant reticulin. Gordon and Sweet. x 125.

activity. A small pararectal lymph node and ten haemorrhoidal lymph nodes showed only reactive changes. A diagnosis of Hodgkin's disease was made.

### DISCUSSION

**Incidence.** Secondary involvement of the rectum in Hodgkin's disease is uncommon (Spiesman and Rubenstein, 1942; Gechman et al., 1956; Shapiro, 1961; Scheffer and Hofstede, 1965). Primary rectal involvement is rarer still and only a few such cases are on record. Spiesman and Rubenstein (1942) referred to a case of primary Hodgkin's disease of the rectum observed by B. Nieman but gave no details. A single case was included in Warren and Lulenski's (1942) series of primary intestinal lymphomas, including 13 cases of Hodgkin's disease: a polypoid rectal lesion, 3cm in diameter, was removed from a patient of unstated sex and age who survived at least 2 years. Similarly, Allen et al. (1954) included a case of primary rectal Hodgkin's disease amongst 9 cases of primary lymphoma of the large bowel and 11 cases of primary Hodgkin's disease of the gastro-intestinal tract, but gave no clinical details. It is evident that none of their cases involving colon and rectum survived for 5 years. A single case of primary rectal Hodgkin's disease was present amongst the pooled material of several London medical schools (Dawson et al., 1961; Cornes, 1967). This was a woman aged 53 years who survived resection and radiotherapy for at least 1 year and 5 months. A similar case of primary Hodgkin's disease of the rectum is included in the series reported by Perry et al. (1972) and is presumed to be the present case. In view of the extreme rarity of primary Hodgkin's disease of the rectum and the limited record of Hodgkin's disease with secondary rectal involvement the salient features will be briefly summarized.

**Clinical features.** The age and sex incidence of primary intestinal (including rectal) lymphomas appears to correspond broadly to that of lymphomas at the more usual sites (Gechman et al., 1956; Dawson et al., 1961; Perry et al., 1972). Intestinal Hodgkin's disease may be accompanied by general symptoms and signs such as malaise, lassitude, loss of weight, loss of appetite and anal pruritus. More specifically there may be a change of bowel habits with diarrhoea or constipation, intestinal bleeding, signs of obstruction and, occasionally, intestinal perforation. Spiesman and Rubenstein (1942) observed a tubular stricture. In primary rectal Hodgkin's disease the patient may complain of a sensation of heaviness in the rectum and of rectal discharge of blood and mucus (Pettinari, 1947; present case), but occasionally the rectal tumour is an incidental and unexpected finding (Perry et al., 1972).

**Associated disorders.** Three of the 37 patients with intestinal lymphomas reviewed by Dawson et al. (1961) had also primary carcinomas of the colon or rectum, the lymphomas being either lymphosarcoma or reticulum-cell sarcoma (Cornes, 1960).

An association between malabsorption and intestinal lymphomas has become recognized in recent years (Gough et al., 1962; Cornes, 1967; Harris et al., 1967). Goodwin and Fry (1973) emphasized that lymphoma of the alimentary tract in these patients is a complication of the enteropathy and not the reverse, and they adduced evidence which suggests that patients with gluten enteropathy may have some form of immune incompetence.

In Dawson's et al. (1961) series 7 of the lymphomas were a complication of long-standing chronic ulcerative colitis, and the authors were of the opinion that a relationship between the two disease entities could not be excluded. We also had the opportunity to observe a caecal lymphosarcoma in a male aged 37 years, who had been diagnosed 7 years previously, and treated successfully, for ulcerative colitis. However, to our knowledge, such an association between ulcerative colitis and Hodgkin's disease has not been recorded.

Shapiro (1961) reported a male of 46 years with generalized Hodgkin's disease and secondary rectal involvement. The patient first presented with anal bleeding which was found to be due to anal Bowen's disease. Previously Graham and Helwig (1959) had claimed an association between intraepidermal carcinoma and visceral malignancies, but such a relationship still lacks general acceptance. However, Starke (1972) observed Bowen's disease of the palm of the hand in a female aged 29 years with Hodgkin's disease.

**Macroscopical appearances.** Rectal Hodgkin's disease usually presents as one or more neoplastic plaques involving the submucosa and muscular coats; frequently the overlying mucosa is ulcerated (Cornes, 1967). Hodgkin's disease presenting as a sessile or pedunculated polyp is rare (Warren and Lulenski, 1942; Cornes, 1967). Encircling plaques may result in rectal stenosis (Gechman et al., 1956). Tumour extension may occur by direct spread into the pelvic soft tissues, by retrograde lymphatic involvement, or by horizontal spread in the colonic mucosa or submucosa (Spiesman and Rubenstein, 1942; Scheffer and Hofstede, 1965).

**Histopathology.** Microscopical examination shows the well-known pleomorphic features of Hodgkin's disease. In the present case there were very numerous bizarre multinucleated tumour giant cells. A preponderance of similar giant cells is occasionally seen in Hodgkin's disease and is regarded by some authors as Hodgkin's sarcoma. Thus Rappaport (1966) depicted an axillary lymph node, 7 cm in diameter, with numerous bizarre atypical giant cells, some of which he regarded as bearing a superficial resemblance to megakaryocytes. The patient, a female aged 34 years, was treated with radiotheraphy and cytotoxic drugs and, in spite of recurrences, survived at least 15 years. Evans (1966) illustrated similar microscopical features in a cervical lymph node of a female aged 58 years who died 10 years after diagnosis and 14 years after onset from a presumably unrelated cause. And Schnitzer et al. (1973) illustrated similar features in children.

Rappaport and Evans's cases, as well as the present case, raise the question whether a marked "megakaryocytoid" pattern might signify a better prognosis.

**Treatment and Prognosis.** Because of the rarity of primary Hodgkin's disease of the rectum the most promising lines of treatment have not been firmly established. In the past rectal lymphomas have been treated with radiotherapy, cytotoxic drugs or surgical excision. Perry et al. (1972) stated that the treatment of choice rests between surgical excision and radiotherapy. Cytotoxic drugs, according to Gechman et al. (1956), whilst not changing the ultimate outcome of the disease, may alleviate the patient's symptoms for considerable periods. However, with the success of modern cytotoxic therapy in Hodgkin's disease this view may no longer be tenable, and although in the past all varieties of large bowel lymphoma have had a poor prognosis (Allen et al., 1954; Dawson et al., 1961; Cornes, 1967) it is reasonable to expect that with present-day oncolytic therapy the prognosis of localized colonic, and especially of rectal Hodgkin's disease, will be greatly improved.

To our knowledge the present case is the only patient with primary Hodgkin's disease of the rectum to be alive and free of disease 7 years after surgical excision.

### SUMMARY

A case of primary Hodgkin's disease of the rectum in a woman aged 55 years is reported. An abdomino-perineal excision of the rectum was carried out; no further treatment was given, and the patient is alive and free from disease 7 years postoperatively. To our knowledge this is the longest surviving patient with this rare localization. Histologically the tumour was characterized by the presence of numerous bizarre "megakaryocytoid" tumour giant-cells. The relevant literature is discussed.

### ACKNOWLEDGEMENTS

We are indebted to Professor R. A. Willis for confirming the diagnosis, and to Mr. W. M. Seymour, Chief Technician, for his technical and photographic assistance.

### REFERENCES

ALLEN, A. W., DONALDSON, G., SNIFFEN, R. C. and GOODALE, F. (1954). **Annals of Surgery, 140,** 428. Primary malignant lymphoma of the gastro-intestinal tract.

CORNES, J. S. (1960). **Journal of Clinical Pathology, 13,** 483. Multiple primary cancers: Primary malignant lymphomas and carcinomas of the intestinal tract in the same patient.

CORNES, J. S. (1967). **Proceedings of the Royal Society of Medicine, 60,** 732. Hodgkin's disease of the gastrointestinal tract.

DAWSON, I. M. P., CORNES, J. S. and MORSON, B. C. (1961). **British Journal of Surgery, 49,** 80. Primary malignant lymphoid tumours of the intestinal tract.

EVANS, R. W. (1966). **Histological Appearances of Tumours,** 2nd ed., figs. 283-285. Livingstone, Edinburgh and London.

GECHMAN, E., BLUTH, I. and GROSS, J. M. (1956). **Archives of internal Medicine, 97,** 483. Hodgkin's disease of the rectum.

GOODWIN, P. and FRY, L. (1973). **Proceedings of the Royal Society of Medicine, 66,** 625. Reticulum cell sarcoma complicating dermatitis herpetiformis.

GOUGH, K. R., READ, A. E. and NAISH, J. M. (1962). **Gut, 3,** 232. Intestinal reticulosis as a complication of idiopathic steatorrhoea.

GRAHAM, J. H. and HELWIG, E. B. (1959). **Archives of Dermatology, 80,** 133. Bowen's disease and its relationship to systemic cancer.

HARRIS, O. D., COOKE, W. T., THOMPSON, H. and WATERHOUSE, J. A. H. (1967). **American Journal of Medicine, 42,** 899. Malignancy in adult coeliac disease and idiopathic steatorrhoea.

PERRY, P. M., CROSS, R. M. and MORSON, B. C. (1972). **Proceedings of the Royal Society of Medicine, 65,** 72. Primary malignant lymphoma of the rectum (22 cases).

PETTINARI, V. (1947). **Chirurgia, 2,** 121. Granuloma maligno a sede rettale.

RAPPAPORT, H. (1966). **Tumours of the Hematopoietic System.** Atlas of Tumor Pathology, Section III, Fascicle 8, figs. 176-177. Armed Forces Institute of Pathology. Washington D.C.

SCHEFFER, E. and HOFSTEDE, D. P. (1965). **Acta medica Scandinavica, 177,** 577. Diffuse Hodgkin's disease of the gastrointestinal tract. Report of a case with profuse steatorrhoea.

SCHNITZER, B., NISHIYAMA, R. H. HEIDELBERGER, K.P. and WEAVER, D. K. (1973). **Cancer, 31,** 560. Hodgkin's disease in children.

SHAPIRO, H. A. (1961). **Archives of internal Medicine, 107,** 270. Primary Hodgkin's disease of the rectum.

SPIESMAN, M. G. and RUBENSTEIN, H. I. (1942). **Annals of internal Medicine, 17,** 349. Hodgkin's lymphogranuloma (rectal stricture). Report of a case.

STARKE, W. R. (1972). **Cancer, 30,** 1315. Bowen's disease of the palm associated with Hodgkin's lymphoma.

WARREN, S. and LULENSKI, C. R. (1942). **Annals of Surgery, 115,** 1. Primary solitary lymphoid tumors of the gastro-intestinal tract.

## Figures and Tables

**Fig. 1. f1:**
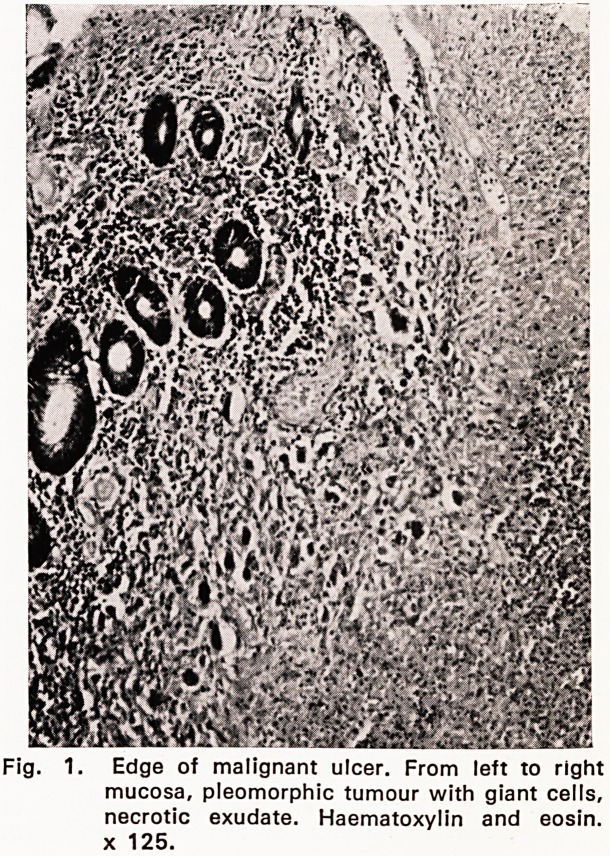


**Fig. 2. f2:**
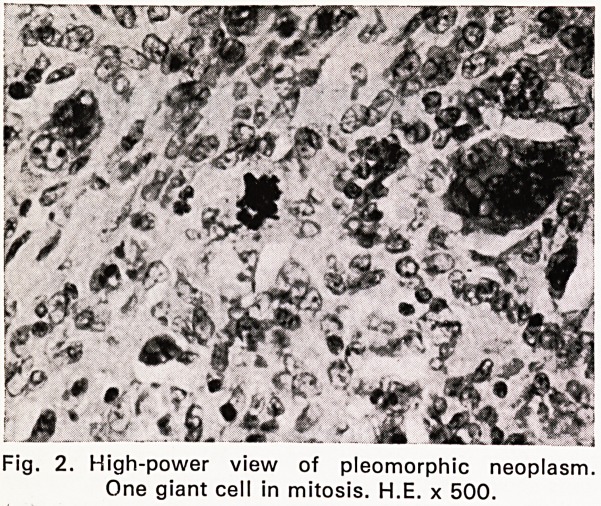


**Fig. 3. f3:**
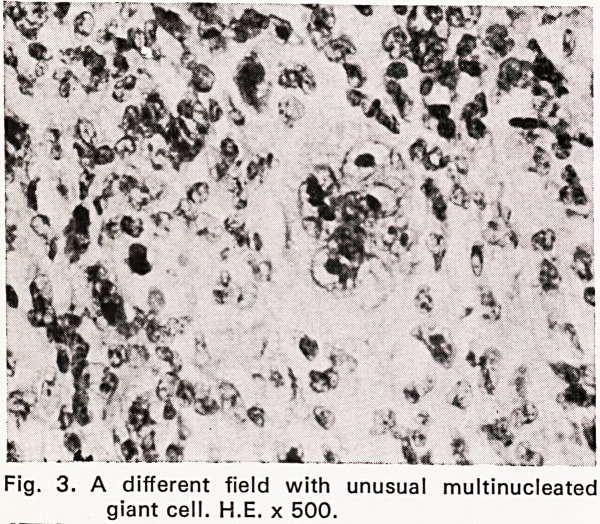


**Fig. 4. f4:**